# The Association Between Relationship Orientation, Relationship Quality and Sexual Satisfaction

**DOI:** 10.1007/s41042-022-00076-0

**Published:** 2022-09-10

**Authors:** Philipp Yorck Herzberg, Swetlana Wildfang, Janine Quittschalle

**Affiliations:** 1 Personality Psychology and Psychological Assessment, Faculty of Humanities and Social Sciences, Helmut-Schmidt-University/University of the Federal Armed Forces Hamburg, Holstenhofweg 85, 22043 Hamburg, Germany; 2 Europäische Fernhochschule Hamburg/University of Applied Science, Hamburg, Germany

**Keywords:** Relationship orientation (short- and long-term), Sociosexuality, Relationship quality, Expectancy fulfilment, Sexual satisfaction, Dyadic data analysis

## Abstract

Although, uncommitted dating via online apps is widespread, most people find value in long-term, trusting relationships. From a social and evolutionary point of view, it has been theorized that mating strategies, and, in particular, short-term strategies make some relationships more vulnerable than others. In our study, we examined short- and long-term relationship orientation and their association with relationship quality. We analysed data from 395 heterosexual couples using the actor-partner-interdependence model in order to explore effects on individuals and couples. Results demonstrated that short-term orientation was associated with lower levels of relationship quality and an increased likelihood of complaints about the partner and the relationship. Long-term relationship orientation, on the other hand, was associated with higher levels of relationship quality. In addition, higher levels of sexual satisfaction mediate the association between short-term orientation and relationship quality. In-depth analyses revealed gender- and couple effects.

## Introduction

The use of Tinder and other online dating apps is on the rise (Hallam et al., 2018; Rosen et al., 2008). Buzzwords most closely associated with Tinder are sex or hook-up apps (Hallam et al., 2018; Sumter et al., 2017). Overall, online dating apps like Tinder claim to promote more short-term relationships (Schwarz et al., 2020). Nevertheless, there still seems to be a desire to start stable and committed relationships. Long-term relationships are highly valued because the need for belonging is a strong, basic mental, and extremely widespread motivation, whilst the absence of belonging has been associated with a number of negative effects on health and well-being (Baumeister & Leary, 1995).

### Mate Choice Strategies: Sociosexual Orientation and Relationship Orientation

Several approaches exist to study which factors lead to more vulnerable or stable relationships. From a social and evolutionary perspective it has been discussed that certain mating strategies make some relationships more vulnerable than others (e.g. French et al., 2019). For example, the well-studied sociosexuality approach assumes that people differ in the constraints they impose on their sexual relationships. In particular, sociosexual unrestricted individuals (vs. restricted individuals) are more motivated to engage in uncommitted sex (Simpson & Gangestad, 1991). Sociosexuality seems to be closely linked to short- (desire for sexual variety) and long-term mating strategies (desire to engage in long-term committed relationships). More unrestricted sociosexually orientated individuals tend to prefer short-term relationships (Simpson & Gangestad, 1991), while restricted sociosexually orientated individuals are more likely to seek long-term relationships. Jonason and colleagues (Jonason et al., 2009; Jonason & Buss, 2012) also emphasize unrestricted sociosexual orientation as an indicator for short-term mating strategies. However, over the years three main criticisms emerged regarding the measurement of sociosexuality: behavioral and attitudinal factors are confounded, underlying one-dimensional structure, and conceptual overlap between biological sex and sociosexuality (for an overview, see Schwarz et al., 2011; Schwarz et al., 2020). Therefore, some researchers have proposed a more multidimensional approach to sociosexuality (Banai & Pavela, 2015; Figueredo & Peñaherrera-Aguirre, 2021; Jackson & Kirkpatrick, 2007), assuming that short-term and long-term mating strategies operate relatively autonomously, although, not completely independent of each other (Figueredo & Peñaherrera-Aguirre, 2021). Similarly, Schwarz & Hassebrauck, (2007) also state that short- and long-term mating strategies operate independently and propose the relationship orientation approach, where the focus lies on preferences instead of behaviors for short- or long-term mating. This is based on the assumption that, for example, a person may be short-term oriented and wants to have many sexual partners but is unable to translate this preference into appropriate behaviors. Previous research showed that high short-term orientation was associated with an unrestricted sociosexuality, a playful love style (Ludus^1^) and a hedonistic present perspective (Schwarz & Hassebrauck, 2007). On the contrary, long-term relationship orientation appears to be related to family orientation, which includes items such as friendly, understanding, creative, domestic, reliable, and was endorsed by individuals who described themselves as long-term oriented (Schwarz et al., 2020). Furthermore, long-term oriented individuals place more value on similarity and a socially responsive personality (Regan et al., 2000), and prefer later rewards to immediate ones (Schwarz, 2008). Gender differences are well documented in research and seem to be stable across decades and cultures (Schwarz et al., 2020). Accordingly, women are more likely to be long-term oriented while men reported that they are more short-term oriented (Figueredo et al., 2005; Figueredo & Peñaherrera-Aguirre, 2021; Hallam et al., 2018; Schwarz et al., 2020; Schwarz & Hassebrauck, 2007).

### Mating Strategies and Couple Effects

At the dyadic level, previous research has shown that differences in motivation for human mating and differences in willingness to engage in sex without attachment and commitment appear to be equally related to partnership disputes and dissolutions (French et al., 2019) as well as a significant mediator of relationship dysfunction (Foster et al., 2006). For instance, unrestricted individuals report lower commitment (Simpson & Gangestad, 1991), fewer relationship-maintenance motivations (Jones, 1998) and more flirtatious advances toward alternative mates (Penke & Asendorpf, 2008). They also perceived more negative interactions in their current romantic relationships and less sexual interest in their partners (Hebl & Kashy, 1995). Sociosexually unrestricted individuals report increased attention to attractive alternative mates (McNulty et al., 2018) and are more likely to be poached by a new romantic partner (Foster et al., 2014). By and large, the extant literature suggests that unrestricted sociosexuality may undermine processes inherent to long-term relationship maintenance which negatively impacts intimates’ relationship satisfaction and long-term stability (French et al., 2019; Penke & Asendorpf, 2008; Rodrigues et al., 2017; Webster et al., 2015). One reason for this could be that expectations regarding various relevant relationship dimensions (i.e., emotional intimacy, sexual intimacy, marital conflicts, intergenerational relationships, and complaints about partner’s lifestyle, Lee 2021) are not met when the relationship is ongoing. According to Sabatelli and colleagues (Sabatelli, 1984; Sabatelli & Cecil-Pigo, 1985; Sabatelli & Pearce, 1986), the fulfillment or non-fulfillment of expectations is related to the number of complaints about the partner or the relationship and often serves as an indicator of relationship quality. Individuals who rate their relationships more positively and express fewer complaints are more likely to be committed to their relationships and perceive their partners as more equal (Sabatelli, 1984; Sabatelli & Cecil-Pigo, 1985; Sabatelli & Pearce, 1986). In addition, lower marital quality is associated with higher levels of verbal aggression (Gavazzi et al., 2000). Similarly, the Marital Comparison Level Index (MCLI, Sabatelli 1984) was found to be a predictor of avoidance strategies for conflict resolution (Marchand & Hock, 2000). A Chinese study found an association between meeting expectations and use of counseling services due to marital problems (Shek et al., 1993).

### Effects of Sexuality and Relationship Duration

Sexual factors such as sexual satisfaction or frequency of sex appear to buffer the negative outcomes of one’s own sociosexuality or that of the partner. French (2019) demonstrated that relatively unrestricted (compared to restricted) sociosexuality was associated with less marital satisfaction at the beginning and a steeper decline in satisfaction over time. According to the authors, relationship dissolution is indirectly predicted by declining marital satisfaction. However, this association was mitigated by frequent sex, high sexual satisfaction and low stress. The results indicated that when someone with unrestricted preferences marries may already experience dyadic stress. However, until now only a few studies examining sociosexual orientation have taken dyadic effects into account (French et al., 2019; Penke & Asendorpf, 2008; Webster et al., 2015). For example, studies have demonstrated that in marriages where at least one of the partner has a more unrestricted sociosexual orientation, there are more worries about infidelity, unfaithfulness and jealous relationship-maintenance behaviors, which could have a detrimental effect on the marriage itself (French et al., 2019; Penke & Asendorpf, 2008; Shackelford et al., 2005).

Moreover, the duration of the relationship also seems to be an important factor. It is well documented that especially with longer relationship duration sexual satisfaction (Fisher, 1987; Quinn-Nilas, 2020; Schmiedeberg & Schröder, 2016) as well as relationship satisfaction (Meltzer et al., 2014) decreases.

### The Present Study

To our knowledge, no study has explicitly examined a multidimensional approach such as relationship orientation on dyadic relationship quality and, in particular, meeting partner expectations on relevant relationship dimensions (i.e., emotional intimacy, sexual intimacy, marital conflicts, intergenerational relationships, and complaints about partner’s lifestyle, Lee 2021). It can be hypothesized that failure to meet partner or relationship expectations may be associated with reduced relationship quality; as the number of complaints increase the relationship becomes more vulnerable (Sabatelli, 1984; Sabatelli & Cecil-Pigo, 1985; Sabatelli & Pearce, 1986). Thus, the present study attempts to fill this research gap. Greater knowledge of factors that help to maintain long-lasting relationships, despite a difficult relationship constellation, not only has theoretical but also practical implications (e.g. counselling). Therefore, the present study aims to examine the following research questions: (1) How is relationship orientation (short vs. long-term) associated with relationship quality in the sense of whether current relationships meet expectations? (2) Are there gender differences in actor and partner effects regarding the association between relationship orientation and relationship quality? (3) Does sexual satisfaction mediate the relationship between relationship orientation and relationship quality? (4) How is relationship duration associated with sexual satisfaction and relationship quality?, and (5) What kinds of couple effects occur? Our primary hypothesis is that short-term (vs. long-term) relationship orientation for either party of an intimate relationship is negatively associated with relationship quality because expectations are less fulfilled (H1). Long-term relationship orientation, on the other hand, is associated with meeting expectations and therefore higher relationship quality (H2). We expect both actor and partner effects for the association between relationship orientation (short-term and long-term) and relationship quality. Furthermore, gender differences in short- and long-term mating strategies are well established (Figueredo et al., 2005; Figueredo & Peñaherrera-Aguirre, 2021; Hallam et al., 2018; Schwarz et al., 2020). For instance, women seem to prefer long-term relationships for enhancement of offspring or physical protection (Buss & Schmitt, 2019; Schwarz et al., 2020), whilst men seem to prefer short-term mating due to reproduction success (produce more offspring, increased fitness) (Buss & Schmitt, 1993). Accordingly, we hypothesize that women (vs. men) prefer more long-term orientated strategies (H3a) and men prefer more short-term relationship orientation (H3b). Furthermore, we explore whether sexual satisfaction mediates the associations between relationship orientation and relationship quality. Consistent with findings that satisfying sexuality is related to sociosexuality and its (negative) marital outcomes (French et al., 2019), we hypothesize (H4a) that sexual satisfaction positively mediates the association between short-term orientation and relationship quality. Regarding long-term orientation, no mediating effect of sexual relationship satisfaction on relationship quality is expected (H4b). Moreover, a number of studies on the association between sexual satisfaction and relationship variables such as relationship quality or relationship satisfaction suggest that more sexually satisfied individuals report higher levels of relationship satisfaction (Christopher & Sprecher, 2000; Sprecher, 2002; Sprecher & Cate, 2004; Yeh et al., 2006). Taking previous research, which shows that sexual satisfaction decreases with relationship duration (Fisher, 1987; Quinn-Nilas, 2020; Schmiedeberg & Schröder, 2016) into account, we expect that the longer the relationship lasts, the lower the sexual satisfaction and relationship quality of short-term oriented individuals (H5). For long-term oriented individuals we did not hypothesize an effect of relationship duration on sexual satisfaction and relationship quality. Given the exploratory nature of these dyadic analyses, we made no a priori predictions regarding possible couple patterns.

## Method

### Recruitment and Participants

847 participants were recruited from two German universities and included students and their parents, relatives and friends. Inclusion criteria required that the couples had been in a heterosexual relationship for at least two months. Questionnaires were handed out in person to each participating couple. A letter accompanying the questionnaires stressed the importance of completing these independently. Participants were instructed to mark all documents with an individual identification code to ensure anonymity and to clearly allocate each participant to a partner. Only couples in heterosexual relationships were included in the study. Additionally, participants with more than 20% missing data were excluded; so were individual questionnaires with no clear partner identification code. These criteria reduced the sample size to 395 couples (790 participants). Data were collected between 2019 and 2020 (before the COVID-19 pandemic).

Female partners had a mean age of 34.1 years (*SD* = 14.4, range = 18–74). On average, male partners were 36.2 years old (*SD* = 15.1, range = 18–80). The mean relationship duration was 10.7 years (*SD* = 12.6, median = 4.0 years) with a minimum of eight months and a maximum of 50 years. Approximately half of the couples (64%) lived together, 12% lived alone, the remainder either with their parents (6%) or in shared accommodation (18%). 37% of the couples were married. The majority of the participants had neither common children (69%) nor children from a previous relationship (91%). 10% had one common child with their partner, 14% two children, and 5% three children. Employed participants totalled 46% in men and 40% in women; whereas 39% of women and 25% of men were students; 6% of women and 8% of men participants were retired; and 3% of women and 3% of men were unemployed.

### Instruments

*Demographics.* A background questionnaire was completed to collect basic demographic information. It includes information on gender, age, status and duration of the current relationship, number and age of children, highest level of education, current profession and income.

*Relationship orientation.* We assessed short- and long-term strategies using the relationship orientation questionnaire (Schwarz et al., 2011; Schwarz & Hassebrauck, 2007, 2015). The questionnaire consists of two scales, each representing the dimensions of long-term orientation and short-term orientation. Participants’ agreement with various statements about themselves and their relationship experiences was recorded on a seven-point scale ranging from (1) “strongly disagree” to (7) “strongly agree.” Examples of these items include: “If the opportunity arises, I would like to have sex with as many people as possible” (short-term relationship orientation) or “Warmth and security are essential components of a relationship” (long-term relationship orientation). Internal consistency was assessed with Cronbach’s α and McDonald’s omega values. Reliabilities for long-term strategies (0.81/0.83) and short-term strategies (0.89/ 0.90) were good. In this sample, the inter-correlation of the scales was − 0.25 (*p* < .001).

*Relationship quality.* To assess couples’ relationship quality, we used the German version (Klann et al., 2003) of the Marital Comparison Level Index (MCLI; Sabatelli 1984). The questionnaire aims to determine the extent to which each partner considers his or her perceptions and expectations of the other or of the relationship to be fulfilled. Thus, a comparison was made between the expected and the experienced quality of the relationship. For each of the 32 items, a 7-point scale ranging from (1) “worse than expected” to (7) “better than expected” was used to indicate the extent to which current experiences in the relationship correspond to expectations of the relationship (Klann et al., 2003). The total score for all 32 items can range from 32 to 224 points. A total score below 128 points indicates disappointment with the partnership. The internal consistency of the test is α = 0.94 and ω = 0.94.

*Sexual satisfaction.* One item was formed to measure sexual satisfaction (“How satisfied are you currently with your sexual relationship with your partner?”). Responses are given on a 7-point scale ranging from (1) “very dissatisfied” to (7) “extremely satisfied”.

*Control variables.* Relationship duration was included as control variable, since the impact of relationship duration on sexual satisfaction is well documented (Quinn-Nilas, 2020; Schmiedeberg & Schröder, 2016).

### Procedure

Our analytic approach was guided by the Actor-Partner Interdependence Model (APIM, Cook & Kenny 2005). According to the APIM, when individuals are involved in a relationship, their outcomes depend not only on their own characteristics and input but also on their partner’s characteristics and input. The APIM estimates these effects which are called actor-effects and partner-effects. Actor-effects are within-person effects: They represent the influence of an individual’s level of a predictor variable on that same individual’s level of an outcome variable. Partner-effects are between-person effects: They represent the influence of an individual’s level of a predictor on his or her partner’s level of the outcome variable. The inclusion of partner effects allows us to test for the hypothesized mutual influence. In addition, the APIM provides estimates of the unique contribution of actor effects controlling for partner effects, and vice versa. Within dyads, we can examine the effects of an individuals’ characteristics on his or her own score, on the dependent variable (actor effect), and the partners’ score on the dependent variable (partner effect).

To test our research questions in respect of the association between relationship orientation (short-term vs. long-term) and relationship quality, as well as gender differences, we conducted an APIM model with distinguishable members. In order to test if gender differences are statistically relevant, a model comparison was performed between a model with distinguishable members and a model with indistinguishable members. This overall test of distinguishability (I-Sat model of complete indistinguishability according to Olsen & Kenny 2006) including long-and short-term relationship orientation, relationship quality, sexual satisfaction and relationship duration as control variable yields a chi-square statistic with 24 degrees of freedom which equals 169.20 (*p* < .01). Because this test of distinguishability is statistically significant, we conclude that members can be statistically distinguished based on their gender. Furthermore, we tested the type of distinguishability by comparing five nested models with progressing constrains on means, correlations and variances to be equal for men and women, starting with a saturated model in which actor- and partner effects are freely estimated for men and women. All model differences tests comparing the different types of distinguishability were statistically significant (*p´s* < 0.05). Furthermore, the Sample Size Adjusted Bayesian Information Criterion was lowest for the model of complete distinguishability (SABIC = 151.52) which indicated that unequal means, unequal correlations and unequal variances described the data best. Given this kind of distinguishability, we computed an APIM model without constrains with long-and short-term relationship orientation predicting relationship quality and controlling for relationship duration. Since the degree of freedom for this model is zero, the chi-square statistic should be zero. Consequently, no probability level can be assigned to the chi-square statistic and no fit indices can be computed. The model converged after 46 iterations. The mediation hypotheses were tested with Actor-Partner Mediation Models (APIMeM) which is an extension of the APIM (Ledermann et al., 2011). The APIMeM allows researchers to estimate mediator effects within dyads, in addition to the effects of an individuals’ characteristics on his or her own score on the dependent variable (actor effect), as well as on the partners’ score on the dependent variable (partner effect). A demonstration of the potential of the APIMeM with a short discussion of methodical issues of meditational testing of dyadic data is given in Sierau & Herzberg (2012). Confidence intervals were computed using percentile bootstrap with 5,000 trials. Model comparisons for gender were computed by setting the corresponding paths equal for females and males and comparing the nested models with the chi-square likelihood ratio tests. Examining whether men and women differ in their actor effects and partner effects was accomplished by setting equality constrains on the corresponding paths and comparing whether a significant χ^2^-change resulted. We also tested dyadic patterns which allows the comparison of dyad member’s respective influence on the outcomes (Ledermann et al., 2011) by comparing the relative size of actor and partner effects (Ledermann et al., 2011). This index is *k*, which is the ratio of the partner effect to the actor effect. The *k* statistic can range from − 1 to 1. Four patterns can be uncovered: Firstly, equal actor and partner effect (couple pattern, *k* = 1); secondly same size, but different signs of actor and partner effects (contrast pattern, *k* = -1); thirdly zero partner effects (actor-only pattern, *k* = 0) and fourthly zero actor effects (partner-only pattern, *k* = 0). Standardized estimates used the pooled variances and the mean difference across members.

## Results

### Descriptive Analyses

Table 1 displays the descriptive statistics and bivariate associations among the main variables. Women reported higher long-term relationship orientation and lower short-term relationship orientation than men (*p´s* < 0.001) (H3a and H3b). Relationship quality was high, 82% of the women and 89% of the men exceeded the cut-off of 128 points of the MCLI. There were no differences in relationship quality and sexual satisfaction between women and men. All correlations between the dyad members were statistically significant at *p* < .001. Length of partnership was significantly negatively related to sexual satisfaction for women (-0.19) and men (-0.20).

**Table 1:** *Descriptive Statistics and Inter-correlations for Study Variables*

	Women		Men										
Variable	M	SD	M	SD	t^a^	*p*	d	1	2	3	4	5	6
1. Long-term relationship orientation	6.32	0.63	6.01	0.77	7.81	< 0.001	0.39	0.38**	− 0.26***	0.15**	− 0.03	0.15**	0.17***
2. Short-term relationship orientation	1.94	1.01	2.41	1.31	-6.85	< 0.001	− 0.35	− 0.20***	0.32***	− 0.23***	− 0.09	− 0.32***	− 0.35***
3. Marital Comparison Level Index^1^	155.41	27.64	156.68	25.75	-0.94	0.367	− 0.05	0.20***	− 0.23**	0.46***	0.32***	0.03	0.05
4. Sexual satisfaction	5.00	1.59	5.03	1.60	-0.32	0.748	− 0.02	0.08	− 0.19***	0.33***	0.52***	− 0.19***	− 0.20***
5. Age	34.1	14.4	36.2	15.1	-11.54	< 0.001	− 0.58	0.12	− 0.29**	0.09	− 0.17***	0.97***	0.90***
6. Length of partnership	10.7	12.6	10.7	12.6	-	-	-	0.16**	− 0.25***	0.07	− 0.19***	0.90***	-

*Note*: Correlations for women are above the diagonal; for men below the diagonal. Correlations between the dyad members are presented in bold along the diagonal. ^a^*df* = 394, ^1^ total score.

*** *p* < .001, ** *p* < .01, * *p* < .05.

### How is Relationship Orientation (short- vs. long-term) Associated with Relationship Quality?

#### Association Between Short-term Relationship Orientation and Relationship Quality

**Actor and partner effects.** For short-term relationship orientation, the actor effect for women was − 0.18 (*p* < .001, 95% CI [-0.25, − 0.09], the overall standardized effect equaled − 0.25. The actor effect for men was − 0.10 (*p* = .002, 95% CI [-0.16, − 0.04] and the overall standardized actor equaled − 0.14. The difference between the two actor effects was statistically not significant (*p* = .174). The overall actor effect was − 0.14 and was statistically significant (*p* < .001, 95% CI [-0.18, − 0.09]. The partner effect from men to women was 0.01, which was not statistically significant (*p* = .690, 95% CI [-0.05, 0.08], and its overall standardized effect was 0.02. The partner effect from women to men is − 0.10 and is statistically significant (*p* = .006, 95% CI [-0.18, − 0.03] and its overall standardized partner effect was − 0.14. When tested if the two partner effects are equal, the difference was statistically significant (*p* = .033, 95% CI [0.01, 0.23]. The overall partner effect of − 0.04 was not statistically significant (*p* = .056, 95% CI [-0.09, 0.00].

**Couple patterns** Next, the relative sizes of the actor and partner effects were considered. Since the standardized actor effects of both women and men were greater than 0.1 in absolute value and were statistically significant, the ratio of the partner effect to the actor effect (k) can be interpreted. The value of k for women equaled − 0.08; the k of the men was equal to 1.03. In order to investigate dyadic patterns in the APIM, a non-parametric bootstrap with 5000 replications was used to calculate the confidence interval of k. For women, it can be concluded that the actor-only model (*k* = 0) was plausible. This can be deduced from the 95% percentile CI which ranges from − 0.45 to 0.42. For men, it can be concluded that the couple model (*k* = 1) was plausible, because the CI ranges from 0.22 to 3.59. Finally, based on the bootstrapped CI of the difference between both k’s, we can also conclude that there was a significant difference between both k’s (*p* = .714, 95% CI [-3.82, − 0.06].

#### Association Between Long-term Relationship Orientation and Relationship Quality

**Actor and partner effects** For the second independent variable, long-term relationship orientation, the actor effect for women was 0.074 (*p* = .335, 95% CI [− 0.08, 0.23] with an overall standardized effect of 0.062. The actor effect for men was 0.17 (*p* = .002, 95% CI [0.06, 0.28] and the overall standardized actor effect for men was 0.14. When tested whether the two actor effects were equal, the difference was found not to be statistically significant (*p* = .339, 95% CI [-0.30, 0.09]. The overall actor effect equaled 0.12 and was statistically significant (*p* = .005, 95% CI [0.04, 0.21]. The partner effect from men to women was equal to 0.14, which was statistically significant (*p* = .018, 95% CI [0.03, 0.26], and its overall standardized effect equaled 0.12. The partner effect from women to men was − 0.05 and was not statistically significant (*p* = .496, 95% CI [-0.19, 0.09] and its overall standardized partner effect was − 0.04. When tested whether the two partner effects were equal, the difference was not statistically significant (*p* = .058, 95% CI [0.00, 0.39]. The overall partner effect of 0.30 was not statistically significant (*p* = .298, 95% CI [-0.04, 0.13].

**Couple patterns** The relative sizes of the actor and partner effects were considered for long-term relationship orientation. The standardized actor effects of both women and men were greater than 0.1 in absolute value and they were statistically significant. The value of k for women equaled − 0.80; the k of the men was to 0.50. Again, a non-parametric bootstrap with 5000 replications was used to calculate the confidence interval of k. For women, it can be concluded that the contrast model (*k* = -1) was plausible since the CI ranged from − 1.91 to -0.15. For men, the CI for k was very wide and it could not be determined which model was most likely. Specifically, the confidence interval ranged from − 1.01 to 2.85. Finally, based on the bootstrapped CI of the difference between both k’s, we can also conclude that there was no significant difference between both k’s (*p* = .379, 95% CI [-4.02, 0.47].

### Does Sexual Satisfaction Mediate the Relationship Between Relationship Orientation and Relationship Quality?

**Figure 1:**
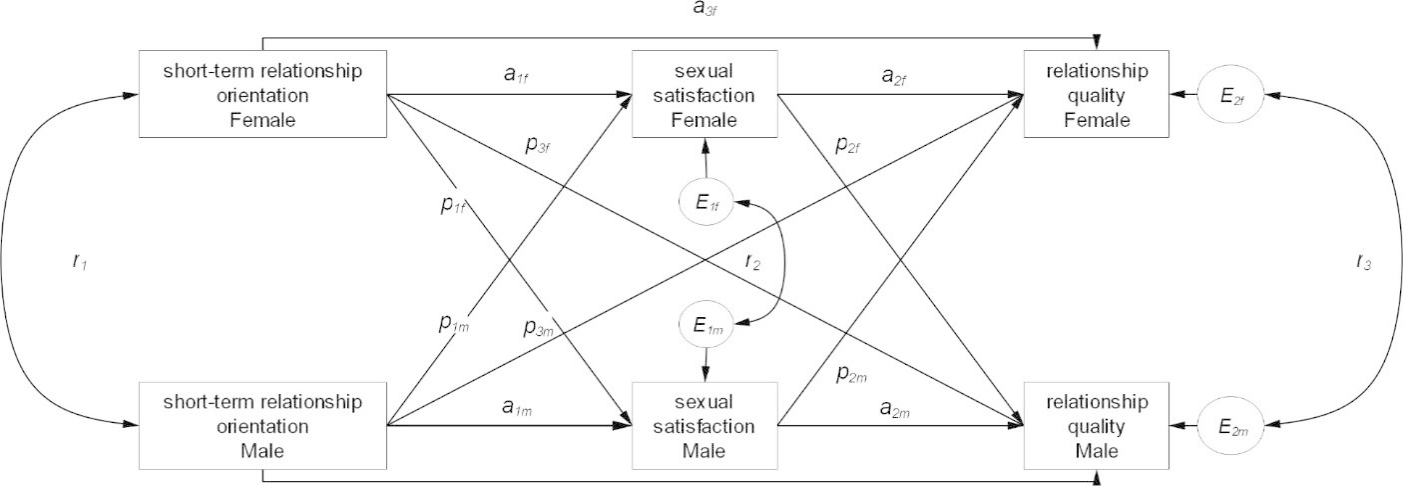
Fig. 1

We addressed whether current sexual satisfaction provided a mediating effect on the relationship between relationship orientation and relationship quality. In order to manage complexity, we computed two independent AIPMeMs, one with short-term relationship orientation as predictor variable and another with long-term relationship orientation as predictor variable. Previously, we tested whether relationship orientation and sexual satisfaction interacted in terms of relationship quality, which would be a violation of the linear mediation model. The RMSEA was less than 0.10 for both relationship orientations (0.000 for short-term and 0.071 for long-term relationship orientation). The chi-square was not statistically significant for short-term orientation ($\chi _{{(df=4)}}^{2}$= 1.10, *p* = .895) but statistically significant for long-term orientation ($\chi _{{(df=4)}}^{2}$= 11.98, *p* = .018). Accordingly there was no evidence of an interaction for short-term but a for long-term orientation. Furthermore, the combined test of mediation was statistically significant ($\chi _{{(df=2)}}^{2}$= 29.96, *p* < .001), with an RMSEA of 0.188 for short-term but not for long-term orientation ($\chi _{{(df=2)}}^{2}$= 0.62, *p* = .735), with an RMSEA of 0.000. The rule that when the RMSEA is greater than 0.10 and the chi-square is statistically significant was evidence that one or more of the indirect effects was nonzero (Kenny, 1996). This was the case for short-term but not for long-term orientation. Therefore, we reported the results of the APIMeM for short-term orientation only. Figure 1 presents the effects in the mediational model. Because the omnibus test indicated no evidence that couple members could be distinguished by sex ($\chi _{{(df=6)}}^{2}$= 8.70, *p* = .191), the dyad members were treated as if they were indistinguishable.

The squared multiple correlation for sexual satisfaction was 0.13 and for relationship quality was 0.37. For ease of interpretation, we reported the standardized estimates computed with the pooled variances. The unstandardized estimates with their standard errors and 95% CI are shown in Table 2.

**Table 2:** Actor and Partner effects for the APIMeM for short-term relationship orientation

Direct actor effects	Estimate	SE	95% CI^a^			Standard Estimate	p
a_1_ (X->M)	− 0.22	0.05	[-0.32, − 0.12]			− 0.16	<. 001
a_2_ (M->Y)	0.13	0.02	[0.09, 0.17]			0.24	< 0.001
a_3_ (X->Y)	− 0.12	0.02	[-0.17, − 0.07]			− 0.17	< 0.001
Direct partner effects							
p_1_ (X->M)	0.07	0.05	[-0.03, 0.16]			0.05	0.155
p_2_ (M->Y)	0.06	0.02	[0.02, 0.10]			0.11	0.004
p_3_ (X->Y)	− 0.04	0.02	[-0.09, 0.00]			− 0.06	0.070
Indirect actor effects							
Total indirect	− 0.025	0.009	[-0.044, − 0.008]			− 0.034	0.003
Actor-Actor indirect	− 0.028	0.008	[-0.046, − 0.014]			− 0.039	< 0.001
Partner-Partner indirect	0.004	0.003	[-0.002, 0.011]			0.005	0.159
Indirect partner effects							
Total indirect	− 0.004	0.009	[-0.023, 0.013]			− 0.079	0.646
Actor-Partner indirect	− 0.013	0.005	[-0.025, − 0.004]			− 0.018	0.004
Partner-Actor indirect	0.008	0.006	[-0.003, 0.021]			0.012	0.155
Covariance							
r_1_	0.43	0.07	[0.29, 0.57]			0.32	< 0.001
r_2_	1.32	0.13.	[1.07, 1.56]			0.54	< 0.001
r_3_	0.23	0.03	[0.16, 0.29]		0.39		< 0.001

*Note*: ^a^ 95% bias-corrected confidence intervals from 5000 bootstrap samples.

The denotations of letters are given in Fig. 1.

Values in bold are significant at *p* < .01.*APIMeM*.

**Short-term orientation and sexual satisfaction** The actor effect of short-term relationship orientation on sexual satisfaction was statistically significant with − 0.16 (*p* < .001), whereas the partner effect was not (0.05, *p* = .155). The ratio of the partner to the actor effect was *k* = -0.29 with a 95% CI from − 0.95 to 0.11. This indicated that the contrast (*k* = -1) and the couple (*k* = 1) models were implausible, and that the actor-only model (*k* = 0) was plausible. The actor effect of sexual satisfaction on relationship quality was statistically significant with 0.24 (*p* < .001), the partner effect was also significant (0.11, *p* = .004). As *K* was 0.44 with a 95% CI from 0.135 to 0.896, it can be concluded that the model lies in between the actor-only (*k* = 0) and the couple (*k* = 1) models. The actor effect of short-term relationship orientation on relationship quality was statistically significant with − 0.17 (*p* < .001), whereas the partner effect was not (-0.06, *p* = .070). Further, the indirect, direct, total indirect, and total direct effects of short-term relationship orientation on relationship quality are reported and presented in Table 3. The total actor effect from short-term relationship orientation to relationship quality was statistically significant with − 0.21 (*p* < .001). The direct effect with − 0.17 was also statistically significant (*p* < .001) and explained 83.24% of the total effect. The total actor indirect effect was statistically significant with − 0.03 (*p* = .003) and explained 16.76% of the total effect. The actor-actor indirect effect was also statistically significant with − 0.04 (*p* < .001) and explained 19.27% of the total effect. The partner-partner indirect effect was not statistically significant with 0.01 (*p* = .159) and explained 2.51% of the total effect.

**Short-term orientation and relationship quality** We proceed with the partner effects from short-term relationship orientation to relationship quality. The total partner effect was not statistically significant with 0.07 (*p* = .052). The direct effect was not statistically significant with − 0.06 (*p* = .070) and explained 90.97% of the total effect. There were two indirect effects. The total partner indirect was not statistically significant with − 0.08 (*p* = .646) and explained 9.03% of the total effect. The actor-partner indirect effect was statistically significant with − 0.02 (*p* = .004) and explained 26.65% of the total effect. The partner-partner indirect effect was not statistically significant with − 0.01 (*p* = .155). Because the total partner effect was not statistically significant, it is inadvisable to examine the percent of total effect that is mediated.

### How is Relationship Duration Associated with Sexual Satisfaction and Relationship Quality?

An APIM with sexual satisfaction, relationship quality and relationship duration was computed; and relationship duration was regressed on sexual satisfaction and relationship quality. Relationship duration was statistically significantly negatively associated with sexual satisfaction for women and men (-0.20, − 0.19 both *p´s* < 0.001, respectively), and statistically significantly associated with men’s relationship quality (0.13, *p* = .006) but not with women’s relationship quality (0.04, *p* = .370).

## Discussion

In the present study, we gathered data from 395 heterosexual couples to examine how short- and long-term relationship orientation and expectancy fulfillment were related to relevant relationship dimensions (i.e., emotional intimacy, sexual intimacy, marital conflicts, intergenerational relationships, and complaints about partner’s lifestyle, Lee 2021). This study is the first to examine underlying dyadic processes by explicitly testing for specific couple patterns (Kenny et al., 2006; Kenny & Ledermann, 2010), thereby allowing us to identify the nature of dependence in dyadic processes. To examine whether relationship orientation (short vs. long-term) is related to relationship quality in the sense of whether current relationships meet expectations, we computed an APIM once with short-term and once with long-term orientation as predictor variables and MCLI as independent variable. The results indicate that short-term orientation is negatively associated with one’s expectancy fulfillment. Long-term orientation, on the other hand, is positively associated with expectancy fulfillment. Thus, hypotheses 1 and 2 were confirmed. However, mainly actor effects were found, which means that short-term oriented (vs. long-term oriented) individuals are less (vs. more) likely to rate their expectations as fulfilled. In contrast to previous studies (Penke & Asendorpf, 2008), no partner effects were found. Thus, our findings complement and extend previous research in which high short-term orientation was associated with unrestricted sociosexuality (Schwarz & Hassebrauck, 2007). Unrestricted sociosexuality, in turn, increases the likelihood of relationship stress and dissolution (French et al., 2019), infidelity in long-term relationships (Penke & Asendorpf, 2008), increased attention to partners outside the couple (McNulty et al., 2018), and flirtatious behavior when meeting an attractive stranger of the opposite sex (Penke & Asendorpf, 2008). On the other hand, long-term relationship orientation seems to be related to family orientation (friendly, understanding, creative, domestic, reliable) (Schwarz et al., 2020). In addition, long-term oriented individuals place more value on similarity and a socially responsive personality (Regan et al., 2000) and prefer later rewards to immediate ones (Schwarz, 2008). These are all characteristics that might be expected to be less likely associated with disappointed expectations. Furthermore, we examined whether there were gender differences in the actor- and partner effects on the association between relationship orientation (short-term vs. long-term) and relationship quality. Our results showed that previous findings on gender differences are replicable; women are more likely to be long-term oriented, while men report being more short-term oriented (Figueredo et al., 2005; Figueredo & Peñaherrera-Aguirre, 2021; Hallam et al., 2018; Schwarz, 2008; Schwarz & Hassebrauck, 2007).Therefore, hypotheses 3a and 3b were confirmed. Focusing on the short-term relationship context, an actor effect was found for both men and women. Moreover, women’s short-term orientation also seems to be related to their partner’s expectancy fulfillment (partner effect). For long-term oriented women, no association was found with the fulfillment of either their own or their partner’s expectations. In contrast, men’s long-term orientation was positively associated with both their own expectation fulfillment (actor effect) and that of their partner (partner effect). This is consistent with Webster et al., (2015), which showed that men’s sociosexual attitudes (compared to women’s) are comparatively more strongly related to relationship outcomes for both sexes.

Interestingly, men’s expectancy fulfillment is associated with their partner’s short-term orientation, whereas women’s expectancy fulfillment is associated with their partner’s long-term orientation. Research on jealousy in romantic relationships may provide a possible explanation. Short-term strategy, or unrestricted sociosexuality, is related to infidelity (Penke & Asendorpf, 2008) as well as jealous relationship maintenance behavior (Shackelford et al., 2005). From an evolutionary psychology perspective, Buss (1992) stated that men and women in heterosexual relationships face different challenges related to reproduction. Accordingly, men should be more affected by their partner’s extradyadic sexuality than by her extradyadic emotional commitment. On the contrary, women should be more upset by the discovery of their partner’s extradyadic emotional involvement than by their partner’s extradyadic sexuality. Thus, men may feel more threatened by their partner’s short-term orientation and the flirting and lower commitment that may accompany it, which negatively affects their expectancy fulfillment in the relationship. For women, on the other hand, their partner’s long-term orientation and its determinants seem to correspond to their desire for security and stability (to enhance the survival of offspring or for physical protection, in terms of the evolutionary perspective) with positive effects on their expectancy fulfillment.

To examine whether sexual satisfaction mediates the relationship between relationship orientation and relationship quality, we computed two independent AIPMeMs, one with short-term relationship orientation as predictor variable and another with long-term relationship orientation as predictor variable and sexual satisfaction as mediator variable. As expected (H4a and H4b), our results revealed a mediator effect on sexual satisfaction exclusively for short-term orientation. Although the main effect of short-term orientation on expectancy fulfillment was the largest at 83%, a significant indirect effect was shown through sexual satisfaction. In other words, short-term oriented individuals who are sexually satisfied are more likely to perceive their expectations as fulfilled and express fewer complaints. This is consistent with the findings of French et al., (2019) who found preliminary evidence that frequent sex and high sexual satisfaction buffer the negative effects of unrestricted sexuality. This could be because short-term oriented men and women focus on sexual attractiveness when evaluating their partner (Regan et al., 2000; Schwarz et al., 2020) or because sexual satisfaction serves as an indicator of the extent to which partners are sexually compatible (French et al., 2019). However, sexual dissatisfaction may increase the likelihood of partner conflict and extradyadic sexuality (Foster et al., 2006, 2014; Jones, 1998; McNulty et al., 2018; Penke & Asendorpf, 2008), which may affect relationship stability or prevent it from occurring in the first place. In addition, we computed an APIM to analyze the association between relationship duration, sexual satisfaction and relationship quality. Similarly, a negative association was found between relationship duration and sexual satisfaction regardless of gender. This suggests that the mediating effect of sexual satisfaction may decrease with relationship duration. However, due to lack of long-term data, causality cannot be established. Our data only permit comparisons between couples, not within couples (Brauer et al., 2022). Though, the length of relationship might be related to relationship quality independent of sexual satisfaction. Our results revealed a positive association between the length of a relationship and relationship quality, but only for men. That is, men from couples reporting larger relationship duration feel more satisfied (in the sense that expectations are met) than men from couples with shorter relationship duration. This is consistent with numerous studies that found that women were less satisfied with marriage than men (Kamp Dush et al., 2008; Stevenson & Wolfers, 2009; Whiteman et al., 2007) and reported lower marital quality on average (Amato et al., 2007). A meta-analysis also found statistically significant but very small gender differences in marital satisfaction between wives and husbands, with wives tending to be more dissatisfied than husbands. However, according to the authors, the difference was attributable to the inclusion of clinical samples. Dyadic analyses, on the other hand, found no sex difference (Jackson et al., 2014). No gender differences in relationship quality were found in our sample either.

Finally, this study aimed to identify underlying couple patterns. Until now, there are few studies that include a couple level analysis. To our knowledge, no couple patterns have yet been reported. Therefore, our analyses were exploratory in nature. For short-term oriented women an actor-only pattern was found. In contrast, a couple pattern was found for short-term oriented men. In other words, the fulfillment of men’s expectations depends on both his own relationship orientation and that of his partner. However, this seems to be the case only for short-term orientation. In the case of long-term orientation a contrasting pattern emerges only for women. This expands the previous explanations and shows the way in which both partners depend on each other. In this context, mediation by sexual satisfaction is of interest. The results suggest only actor effects. Thus, our study builds on and expands findings presented by French et al., (2019) by showing that one partner’s relationship orientation has no direct effect on the other’s sexual satisfaction; it does, however, indirectly effect expectancy fulfillment. Thus, an indirect effect of one partner’s sexual satisfaction on the other’s expectation fulfillment was found. As far as we know only little research has been done on this topic with a study on German women which examined whether meeting expectations predicts sexual satisfaction being the only previous study to date (Prentki, 2009).

### Strength, Limitations and Future Directions

The current study offers two major contributions. Firstly, it extends previous work on sociosexuality and relationship outcomes by considering a multidimensional approach. Secondly, dyadic data and couple pattern analyses support interdependence theory (Kelley & Thibaut, 1978) and suggest that the linkages between relationship orientation and relationship quality are determined by the complex interactions between two partners. However, the results of the current investigation should be viewed in light of several limitations. Due to the cross-sectional nature of the study, it is not possible to draw conclusion about long-term effects and causality. Further, the recruitment of the study sample has some limitations, therefore, a selection bias in observational date cannot be excluded. This, as well as the relative homogeneity of the sample, may limit the generalizability of our results. In this context, it should also be noted that couples per se are more likely to be long-term orientated than short-term orientated because they have already entered into a relationship and be committed to their partner. Another limitation is that the current study is based on self-reported measures; therefore, influences such as social desirability cannot be excluded. As relationship duration seems to be associated especially with the mediating variable sexual satisfaction, longitudinal designs would be of great benefit in order to examine changes in the variables over time, as well as to address questions of causality. Since there is recent research emphasizing that intrasexual differences in mating strategies are more, or at least equally, important as intersexual differences (Hallam et al., 2018; Schwarz et al., 2020), further studies should include within-sex differences. In this context, some conceptual overlap between the constructs relationship orientation, sex and expectation fulfillment should be considered, which may result in a significant amount of shared variance. Although, sexual satisfaction seems to mediate negative associations of short-term orientation on relationship quality, other factors (i.e. stress, French et al., 2019) should be included in order to identify protective factors. In addition, it might be interesting to consider differences in couples’ relationship orientations, e.g., congruence or discrepancies in partners’ mating preferences. It is conceivable that short-term orientation is not related to lower satisfaction when both partners are short-term orientated and make arrangements that allow them to express their preferences (e.g. in an open relationship). For instance, previous research on sociosexuality has shown that relationship agreement moderates the negative association between unrestricted sociosexuality and relationship quality, independent of gender (Rodrigues et al., 2017). Studies on dyadic data have examined whether there are congruencies or discrepancies in relationship aspects, e.g., using polynomial regression analyses (Quittschalle & Herzberg, 2017). Further research should address this issue.

### Conclusion

Long-term relationships are highly valued (Baumeister & Leary, 1995). From a social and evolutionary perspective, certain mating strategies, make some relationships more vulnerable than others (e.g. French et al., 2019). All in all, the extant literature suggests that short-term orientation (or unrestricted sociosexuality in terminology of the sociosexuality approach) may undermine processes inherent to long-term relationship maintenance, thereby negatively impacting intimates’ relationship satisfaction and long-term stability (French et al., 2019; Penke & Asendorpf, 2008; Rodrigues et al., 2017; Webster et al., 2015). However, sexual aspects of the relationship appear to mitigate the negative outcomes of sociosexuality. However, until now only a few studies have been examining a multidimensional approach of sociosexual orientation or taking dyadic effects into account (French et al., 2019; Penke & Asendorpf, 2008; Schwarz et al., 2020; Webster et al., 2015). Thus, the present study attempts to fill this research gap, providing evidence that short-term and long-term relationship orientations are differently associated with relationship quality. Besides actor- and partner-effects, couple effects were also found. Moreover, our results confirm previous evidence that sexual aspects mediate negative outcomes, especially meeting expectations within the relationship. This is of particular interest because a better knowledge of factors contributing to the maintenance of long-term relationships has not only theoretical but also practical implications (e.g. in counselling, therapy).

## Data Availability

Data can be obtained by request from the first author.

## Footnotes

1 The opinion that Ludus style reflects a playful approach to love has been challenged because it does not refer to indicators of playfulness. Rather, it seems to reflect a Casanova-like approach to relationships characterized by infidelity and extra-dyadic relationships rather than a playful style of love (Proyer et al., 2018).
